# Sodium (^23^Na) ultra-short echo time imaging in the human brain using a 3D-Cones trajectory

**DOI:** 10.1007/s10334-013-0395-2

**Published:** 2013-07-31

**Authors:** Frank Riemer, Bhavana S. Solanky, Christian Stehning, Matthew Clemence, Claudia A. M. Wheeler-Kingshott, Xavier Golay

**Affiliations:** 1 NMR Research Unit, Department of Neuroinflammation, Queen Square MS Centre, UCL Institute of Neurology, Queen Square, London, WC1N 3BG UK; 2 Philips Research Hamburg, Philips Healthcare, Hamburg, Germany; 3 Philips Clinical Science Group, Philips Healthcare, Guildford, UK; 4 Department of Brain Repair and Rehabilitation, UCL Institute of Neurology, Queen Square, London, UK

**Keywords:** Sodium, ^23^Na, UTE, Non-Cartesian, Cones

## Abstract

**Object:**

Sodium magnetic resonance imaging (^23^Na-MRI) of the brain has shown changes in ^23^Na signal as a hallmark of various neurological diseases such as stroke, Alzheimer’s disease, Multiple Sclerosis and Huntington’s disease. To improve scan times and image quality, we have implemented the 3D-Cones (CN) sequence for in vivo ^23^Na brain MRI.

**Materials and methods:**

Using signal-to-noise (SNR) as a measurement of sequence performance, CN is compared against more established 3D-radial *k*-space sampling schemes featuring cylindrical stack-of-stars (SOS) and 3D-spokes kooshball (KB) trajectories, on five healthy volunteers in a clinical setting. Resolution was evaluated by simulating the point-spread-functions (PSFs) and experimental measures on a phantom.

**Results:**

All sequences were shown to have a similar SNR arbitrary units (AU) of 6–6.5 in brain white matter, 7–9 in gray matter and 17–18 AU in cerebrospinal fluid. SNR between white and gray matter were significantly different for KB and CN (*p* = 0.046 and <0.001 respectively), but not for SOS (*p* = 0.1). Group mean standard deviations were significantly smaller for CN (*p* = 0.016). Theoretical full-width at half-maximum linewidth of the PSF for CN is broadened by only 0.1, compared to 0.3 and 0.8 pixels for SOS and KB respectively. Actual image resolution is estimated as 8, 9 and 6.3 mm for SOS, KB and CN respectively.

**Conclusion:**

The CN sequence provides stronger tissue contrast than both SOS and KB, with more reproducible SNR measurements compared to KB. For CN, a higher true resolution in the same amount of time with no significant trade-off in SNR is achieved. CN is therefore more suitable for ^23^Na-MRI in the brain.

## Introduction

### Background

^23^Na-MRI of the brain has been used to show changes in total tissue sodium concentration (TSC) in stroke [1], Alzheimer’s disease [2], Multiple Sclerosis [3–5] and Huntington’s disease [6]. Lesions and pathologies can be detected in these diseases using ^23^Na-MRI, complimentary to information obtained by ^1^H-MRI, as well as statistically significant changes in tissue that appears otherwise normal on ^1^H-images [3–5]. Applications of ^23^Na-MRI are not limited to the brain, changes in the cartilage of the knee that relate to osteoarthritis onset have also been demonstrated [7]. ^23^Na-MRI has therefore the potential to not only become a viable clinical tool to give us new insights into disease mechanisms, but also to monitor the onset and progression of several pathologies.

In comparison with ^1^H-MRI, ^23^Na-MRI suffers in signal detectability due to its lower abundance and smaller gyromagnetic ratio. ^23^Na-MRI studies at 3T therefore require long scans of 10–20 min and large voxel sizes of around 4 × 4 × 4 mm^3^ [3–5], compared to ^1^H-MRI where anatomical images with a 1 × 1 × 1 mm^3^ voxel size are commonly acquired in <10 min at 3T. In addition, the spin 3/2 properties of the sodium nucleus yield a bi-exponential longitudinal (*T*_1_) and transverse decay (*T*_2_) in biological tissue [8]. The fast component of the *T*_2_-decay is the predominant decay mode (~60 % of the signal), with a corresponding relaxation time that has been reported to be as short as 0.5 ms in cartilage of the knee [9], and to be around 2–5 ms in the human brain [10]. Therefore, to capture the entirety of the sodium signal, it is necessary to use ultra-short echo time (UTE) acquisition strategies, featuring radial or spiral free induction decay (FID) *k*-space sampling, rather than spin or gradient echoes as commonly used in ^1^H-MRI. As a result, UTE sampling increases the signal-to-noise ratio (SNR) significantly over standard spin and gradient echo sequences. The increase in SNR can in turn be used to shorten scan times or to achieve higher resolutions.

Nevertheless, UTE acquisition methods are generally slow compared to echo sampled MRI and efficient sampling of *k*-space is an important factor in achieving high SNR and resolution in an acceptable scan-time. For ^23^Na-MRI acquisitions, radial and spiral UTE sequences are not prone to water-fat shift artifacts, but suffer from other more complex artifacts: off-resonance signal components, for example, do not lead to shift or ghost artifacts, but result in blurring, as *k*-space is sampled in a center-out manner [11, 12]. Furthermore, *k*-space undersampling can result in streaking artifacts or, more commonly, aliasing patterns, which are most notable as non-uniform background noise or fold-over like repetitions of the image.

Many different sequences exist that aim to maximize efficiency of *k*-space sampling such as stack-of-stars (SOS) [13], 3D-spokes [14] (such as kooshball (KB) sampling), twisted-projection-imaging (TPI) [15], 3D-Cones [16] and single-point ramped imaging with *T*_1_ enhancement (SPRITE) [17]. Romanzetti et al. [18] have performed a comparison of these readouts for ^23^Na-imaging in a short study on an agarose phantom at 9.4T and found the 3D-Cones sequence to perform best. Whilst interesting, these results are difficult to translate to the human case, due to the use of high field strength and the lack of consistency between protocols (in terms of repetition time (TR), voxel size, flip angle (FA) and receiver bandwidth (BW)). Therefore, here we set out to implement and compare the 3D-Cones sequence to two other in vivo protocols, varying only sampling density and fixing image contrast parameters such as TR and FA, for a given scan duration of 18 min on a 3T clinical system. Theoretical resolution was separately assessed using a numerical phantom.

### Sequences

Both the SOS and KB sequences that are described in the following are available on our Philips clinical MR-system through a research agreement as a non-commercial option, and can easily be adapted for ^23^Na-MRI. CN was implemented separately by the authors.

#### Stack-of-stars (SOS)

**Fig. 1:**
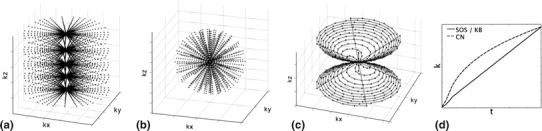
From left to right, illustration of the sampling schemes: **a** radial SOS, **b** radial KB, **c** CN. Evolution of the trajectory in terms of distance from the center of *k*-space (*k* = 0) against time for one readout is shown in **d**)

This sequence is a 3D sequence with an in-plane “2D” radial sampling using Cartesian slice encoding [13]. The *k*-space sampling for each plane corresponds to a star-like shape where points are collected from the center of *k*-space outwards along straight lines (Fig. 1a). Points are sampled at regular intervals along each projection line. A volume can be visualized as a cylindrical SOS. The downside of this acquisition is that *k*-space is non-uniformly sampled, with a higher concentration of points around the *k*-space origin, and sparse sampling at higher frequencies away from the origin. SOS however, using planar section acquisition, allows the use of non-isotropic volumes, facilitating higher in-plane resolution by using thicker sections, or shorter scan times by reduction of the total number of acquired sections. Sampling of the trajectory begins on the ramp of the readout gradient, with quadratic spacing, after which samples are acquired linearly (Fig. 1d).

#### Kooshball (KB)

In the 3D-spokes radial scheme [14] (Fig. 1b) available on the 3T Philips Achieva scanners, samples are acquired at regular intervals along straight lines similar to SOS, but are not restricted to 2D-planes. *k*-space is explored in 3D with the end points of all readouts distributed along spiral interleaves running on a sphere from one pole to the equator [19].

This trajectory allows UTE imaging with shorter echo times when compared with SOS sampling, as no phase encoding gradients are required. Furthermore, polar undersampling can be performed in all three spatial dimensions with only mild impact on the point-spread-function as demonstrated in ^1^H-MRI cardiac applications, where the number of interleaves can be adapted to the cardiac cycle to avoid motion artifacts [20]. Samples are acquired along each line of *k*-space is identically to those acquired in the SOS sequence in terms of sampling rate and evolution (Fig. 1d).

#### 3D-Cones (CN)

Another trajectory that can be exploited for UTE sampling of *k*-space is that of spirals. There are different ways to collect spiral trajectories in 3D, a popular one is to twist the spiral arms around a conical volume (Fig. 1c). The most well known conical sampling strategies are TPI [15] and CN [16]. TPI has been developed specifically for ^23^Na-MRI and to optimize SNR, typical readout lengths for TPI are of the order of ~20 ms, therefore making it difficult to be compared to other sequences for the same readout length. The CN trajectory produces similar waveforms to TPI, but has been developed for rapid- and UTE-^1^H-imaging. CN waveforms can easily be generated for a given resolution and field-of-view (FOV) by code available from Gurney et al. [16], and can be adapted to trade-in speed for SNR through signal averaging [21]. Compared to the two radial sequences used here, the CN-trajectory moves away from the center of *k*-space more quickly (Fig. 1d), resulting in less oversampling of central *k*-space, therefore making the trajectory more effective.

In this study, our aim was to assess the performances of the CN sequence in vivo for quantitative ^23^Na in the brain at 3T.

## Materials and methods

### Subjects

Five healthy subjects consented to take part in the study (age 30 ± 8 years, 2 male, 3 female), approved by the local ethics committee.

### MR-protocol

All scans were performed on a 3T Philips Achieva TX (Philips Healthcare, Best), using a single-resonant ^23^Na fixed-tune birdcage coil supplied by Rapid Biomed (Rimpar, Germany).

Two phantoms containing 4 % agar and NaCl at 33 and 66 mM in water were placed on either side of the head on each subject, as commonly used for quantification purposes [3–5].

A 6-min proton-density weighted (PD-w) spin-echo ^1^H scan (resolution 1 × 1 × 2 mm^3^, FOV 240 × 240 × 200 mm^3^, TR = 3,250 ms, TE = 34 ms) was performed before the ^23^Na scan for anatomical reference and registration to the ^23^Na scans to later create the regions of interest (ROIs) for analysis. This was conducted during the same examination using the quadrature body coil to avoid subject repositioning. The T_1_ of ^23^Na at 3T has previously been determined to be between 21 ms (brain white matter) and 47 ms (cerebrospinal fluid) [22] and the TR has therefore been chosen to be TR = 120 ms, also in accordance with previous quantification studies [3–5]. We chose a nominal isotropic voxel size of 4 mm and a nominal field-of-view (FOV) of 240 × 240 mm^2^ for all scans. A readout window of 5.6 ms was used for all scans, based on a calculation to optimize SNR whilst limiting blurring effects in radial sequences by Rahmer et al. [23]. For this calculation, we used the previously measured shortest *T*_2_ values in the brain of around 1.5 ms for the short component, and around 18 ms for the long component [10]. The three ^23^Na-MRI protocols were matched for time, adjusting sampling densities only and run at their minimal echo time. Scans were optimized for a scan time of 18 min. CN scans were acquired on the same volunteers, but in a different session on a different day than KB and SOS. The PD-w images acquired separately for both sessions were used to identify the same anatomical regions on the volunteers.

#### Stack-of-stars (SOS)

Stack-of-stars were acquired after 3D non-selective block excitation (length 321 μs, amplitude 70 μT) using ramp-sampled FID acquisition with a delay of 0.27 ms between the end of the excitation pulse and the start of readout (the UTE echo time, TE) to accommodate the long readout-gradients imposed by the bandwidth. During this delay, a slice selective gradient is applied, after which the radial spokes are acquired. A total of 9,217 readouts were performed, total acquisition time 18 min, 27 s.

#### Kooshball (KB)

Excitation for KB is identical to SOS, but the slice selective gradient is absent, as localization is achieved by varying the amplitude of the readout gradients in 3D, rather than 2D space. Echo time is 0.27 ms as in SOS, due to the chosen readout bandwidth. We used the default setting of 10 interleaves over which to project the radial lines, as cardiac cycle-induced motion was not an issue. In total, 9,240 readouts were performed, total acquisition time 18 min, 30 s.

#### 3D-Cones (CN)

The gradient waveforms for the CN trajectory were generated using the Matlab code available from Gurney et al. [16], using a custom implementation for our scanner. Sinusoidal gradient waveforms are ramped up at maximum slew rate and extended to the maximum gradient strength within the desired readout length to cover *k*-space as spirals on conical surfaces. As spiral sequences explore *k*-space more efficiently [12], four averages were run to match the scan time to that of SOS and KB with a total number of readouts of 9,352, total acquisition time 18 min, 42 s.

Differences in acquisition parameters for the three sequences are summarized in Table 1.

**Table 1:** Summary of differences in acquisition parameters used for the comparison of the three sequences

Sequence	Stack-of-stars (SOS)	Kooshball (KB)	3D-Cones (CN)
Echo time, TE (ms)	0.27	0.27	0.22
Acquisition time (mins.)	18.4	18.5	18.7
No. of readouts	9,217	9,240	9,352 (4 × 2,338)

### Theoretical SNR efficiency

Non-Cartesian sampling schemes are known to suffer from an inherent loss in SNR of around 25 % compared to Cartesian sampling, due to the non-homogenous properties of their sampling point densities, leading to an increase in image noise [24]. A derivation of this can be found in Nagel et al.’s work [24] based on Liao et al.’s work [25] on image space variance for a given sampling density per *k-*space volume. Here, we aim to also include SNR changes due to density compensation in reconstruction, which is performed to account for the high oversampling for small distances to *k* = 0, and undersampling at higher *k*. To compensate for these non-homogenous sampling densities in non-Cartesian sampling, each *k*-space sample is multiplied by a coefficient, a density compensation weight, which is derived from the sampling density itself and which will impact on SNR in the reconstructed image [26, 27].

Signal-to-noise efficiency after reconstruction can therefore be calculated from the density compensation weights:

1 $$ {\text{SNR}}_{\text{efficiency}} \sim \sqrt {\frac{{\left( {\sum\nolimits_{i = n}^{N} {{\text{weights}}(i)} } \right)^{2} }}{{N_{\text{samples}} * {\sum\nolimits_{i = n}^{N} {{\text{weights}}(i)} } ^{2} }}} $$

where SNR_efficiency_ is a number between 0 and 1, 1 corresponding to fully sampled Cartesian sampling, which maximizes SNR. N_sample**s**_ represents the total number of acquisition samples and *i* equals the elements of the density compensation weights, summed between the first and last element *n* to N respectively, which correspond to a set of *k*-space coordinates each.

### Image reconstruction

Stack-of-stars and KB were reconstructed on the scanner using the Philips standard reconstruction code. This was done using a gridding algorithm [28], optimized quadratic weights and a circular (resp. spherical for KB) shutter to exclude artifacts at the edges of the FOV. The CN images were reconstructed offline in Matlab, using Zwart et al.’s [29] iterative weights and Fessler’s [30] min–max non-uniform-fast-Fourier-transform (NUFFT). No *k*-space window-filter functions, such as the SNR-boosting hamming-filter, were used for any of the reconstructions.

### Post-processing and analysis

#### Registration

Registration of the PD-w to SOS, KB and CN scans was performed using SPM8 (University College London, 2011), as it was acquired at a higher resolution, and to later accurately transfer the ROIs to the ^23^Na scans.

#### SNR measures

**Fig. 2:**
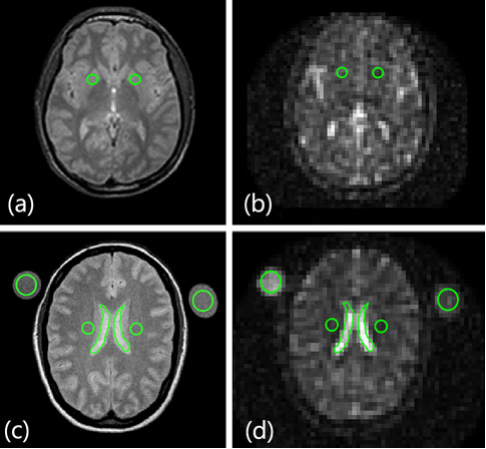
Positions and sizes of the ROIs on ^1^H scan (*left*, **a**, **c**) and transferred onto ^23^Na scan (*right*, **b** ,**d**) for one subject. **a**, **b** Shows the deep grey matter ROIs, **c**, **d** shows the periventricular white matter ROIs as well as CSF and phantom ROIs

Signal-to-noise was measured in a number of areas for each subject. ROIs for intensity measurements where chosen from the ^1^H scans and transferred onto the ^23^Na-MRI scans using OSIRIX (Pixmeo, 2011). Bilateral regions of interest were chosen in periventricular white matter (WM) and in the gray matter (GM) of the putamen. This particular gray matter region was chosen due to its relatively large size and distance from the cerebrospinal fluid (CSF), as compared to cortical GM, to avoid partial volume contamination. Two ROIs of CSF contained in the ventricular spaces were also taken for each volunteer. Single measurements of the phantoms were recorded, as well as measurements outside of the brain to estimate noise. Position and sizes of ROIs are shown in Fig. 2. The SNR for each ROI was calculated as previously described by Qian et al. [31]. This approach is based on Henkelman’s work on measuring SNR at low levels from magnitude images using a quadrature coil [32]. The SNR in this case can then be calculated as:

2 $$ {\text{SNR}} = \frac{{\sqrt {I_{1}^{2} - (\frac{{I_{0} }}{1.25})^{2} } }}{{\frac{{I_{0} }}{1.25}}} $$

where *I*_1_ is the mean intensity over the region of interest and *I*_0_ is the mean intensity of a similarly sized region outside of the brain, used as a noise estimate. To minimize bias due to non-homogenous noise regions, four regions were averaged, taken from each image quadrant. ROIs were circular for WM, GM and phantom measurements, and hand-drawn polygons for the CSF ROIs. WM and GM ROIs contained four voxels making up a volume of approximately 0.26 cm^3^, whereas phantom ROIs contained nine voxels making up a volume of approximately 0.58 cm^3^. CSF ROIs contained at least six voxels and at most ten voxels each, corresponding to approximate volumes of 0.38 and 0.64 cm^3^ respectively. Positioning and size of the ROIs is illustrated in Fig. 2.

#### Statistical analysis

Statistical analysis was performed in Matlab (the MathWorks, 2010) using the statistics toolbox functions for balanced one-way analysis of variance (ANOVA) using Tukey’s honestly significant difference criterion for multiple comparisons. A *p* value of <0.05 was considered statistically significant. *p*-Value statistics for all SNR values for each region were compared for significant differences between the sequences. Significant difference statistics between the WM and GM ROIs, as well as the phantoms were also computed as a measure of contrast, as ^23^Na concentration differ approximately 10 mM between WM and GM [3–5], and 33 mM between the two phantoms. Mean and standard deviations were also computed and medians were used for boxplots. Inter-subject variation was assessed by comparing the standard deviations of the group mean intensities.

#### Resolution measures

Given the different *k*-space trajectories, the effect of the sampling scheme on the point-spread-function (PSF) was assessed for each protocol. A larger PSF effectively means more blurring of the image due to a larger effective voxel size, thus an effective decrease in the resolution. This blurring may also cause the effective voxel size to be different and account for changes in SNR. Therefore, to find the theoretical resolution, the PSF was simulated for each protocol. The PSFs were evaluated offline in Matlab (the MathWorks, 2010) by replacing the *k*-space data with a unity signal and reconstructing it using the same trajectories and density compensation weights as in the in vivo images. Inverse Fourier-transform was performed using Fessler’s min–max NUFFT algorithm [30]. PSFs were normalized and windowed to the same percentage. For KB and CN individual slices were summed up to illustrate the effect of the PSF variation along the *z*-direction. For SOS, this was not necessary as the images are reconstructed slice-wise. No circular or spherical FOV shutters were used for the PSF reconstructions. For comparison, a volume of 60 × 60 × 60 unity matrix representing a Cartesian dataset was inverse Fast-Fourier transformed using the Matlab library function. Effective voxel volumes were determined for the three different *k*-space sampling schemes based on the widening of the PSF in the *x*, *y* and *z*-direction compared to the Cartesian dataset at full width at half maximum (FWHM). For expected brain *T*_2_ values of around 3 ms and 20 ms for the short and long components respectively [10], short *T*_2_-blurring effects were minimal (not shown) for the echo and readout time used and were therefore not included in the analysis.

In addition, spatial resolution in the *x*, *y*-plane was estimated from acquired images on a cylindrical tube filled with water suspended in a saline solution of NaCl (concentration ≈154 mM NaCl). The diameter of the tube was 2.9 cm and the same sequence protocols as used in vivo were used. In agreement with a previous study by Qian et al. [31], signal profiles were taken half-way down the phantom, which can be used to experimentally approximate the FWHM of a symmetric point spread function [11, 31]. As the transition from saline to water is a sharp step-like function, the number of pixels constituting the step in the reconstructed image was measured to estimate resolution.

## Results

### Theoretical SNR efficiency

Theoretical SNR efficiency, as calculated from the density compensation weights, was found to be 87 % for SOS, 91 % for KB and 93 % for CN, compared to the SNR of a homogenously sampled Cartesian acquisition.

### SNR and contrast

**Fig. 3:**
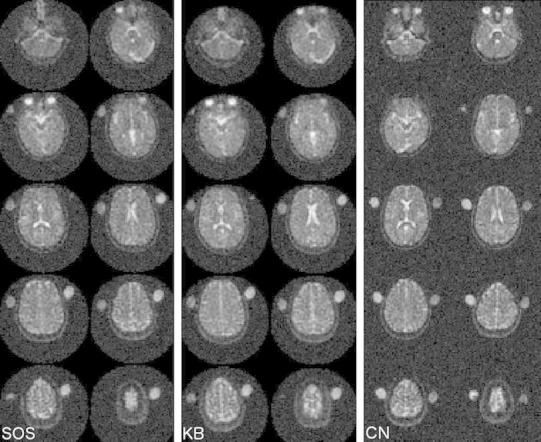
Ten out of the acquired sixty transverse slices for one subject: SOS (*left*), KB (*middle*) and CN (*right*). *Note* CN was acquired on a different day to SOS and KB and the agar phantoms were swapped *left*–*right*

Assorted images for one subject for the different sequences are shown in Fig. 3. Upon close visual inspection, KB is more homogenous but blurrier than both SOS and CN. CN has the least blurring and high intensity regions such as the ones rich of CSF are crisper and better defined. Contrast between different tissue types appears also strongest for CN.

Signal-to-noise (SNR) for each sequence is given in Table 2, for each volunteer, in addition to the mean and standard deviations (SD) in GM, WM, CSF and the two phantoms. SNR differences were not found to be statistically significant for the three sequences (*p* > 0.05), albeit SNR results for SOS were generally lower: SNRs for GM were measured to be of 7.3 ± 1.1 AU for SOS, 8.6 ± 1.3 AU for KB and 7.6 ± 0.3 AU for CN. WM SNRs were 5.8 ± 1.5 AU, 6.6 ± 1.4 AU and 6.5 ± 0.3 AU for SOS, KB and CN respectively. The SNRs found in CSF and the phantoms show a similar trend, as shown in Table 2.

**Table 2:** Table summarizing SNRs found for the three sequences for the individual subjects

SNR ± SD	Stack-of-stars (SOS)					Kooshball (KB)					3D-Cones (CN)				
	GM	WM	CSF	33 mM	66 mM	GM	WM	CSF	33 mM	66 mM	GM	WM	CSF	33 mM	66 mM
Subject 1	6.3 ± 0.1	6.1 ± 0.1	17.4 ± 3.8	6.1	10.1	9.1 ± 0.4	5.5 ± 0.1	15.5 ± 0.8	6.2	10.5	7.5 ± 0.4	6.9 ± 0.1	18.2 ± 0.1	6.6	12
Subject 2	8 ± 0.6	5.7 ± 0.4	17 ± 3.5	6.5	11.6	10.5 ± 0.5	8.1 ± 0.1	23 ± 3.1	7.7	14.4	8 ± 0.1	6.1 ± 0.1	18.2 ± 4.2	7.2	12.5
Subject 3	6.9 ± 0.2	4.4 ± 0.2	15.5 ± 0.9	5.7	9.5	7.9 ± 0.5	5.6 ± 0.1	14.5 ± 3.1	5.8	9.8	7.2 ± 0.4	6.2 ± 0.2	17.7 ± 1	6.4	11.4
Subject 4	8.9 ± 0.2	8.1 ± 0.2	18.3 ± 0.9	8.4	15.5	8.5 ± 0.6	8.2 ± 0.2	21.4 ± 3.7	8.7	13.8	7.7 ± 0.1	6.6 ± 0.4	17.5 ± 0.1	7.3	13.5
Subject 5	6.5 ± 0.2	4.7 ± 0.1	14.8 ± 1	5.5	9.6	7.1 ± 0.1	5.6 ± 0.7	15.3 ± 0.1	8	14	7.7 ± 0.4	6.5 ± 0.3	17.8 ± 0.2	7	12
Mean	7.3 ± 1.1	5.8 ± 1.5	16.6 ± 1.4	6.4 ± 1.2	11.3 ± 2.5	8.6 ± 1.3	6.6 ± 1.4	17.9 ± 3.9	7.3 ± 1.2	12.5 ± 2.2	7.6 ± 0.3	6.5 ± 0.3	17.9 ± 0.3	6.9 ± 0.4	12.3 ± 0.8

Last row gives the mean values for the cohort. SNR was calculated correcting for low SNR magnitude images. Generally highest SNR occurs for the KB scans, but is not statistically significantly different from SOS or CN. Lowest standard deviations and inter-subject variability has been found for the CN sequence

**Fig. 4:**
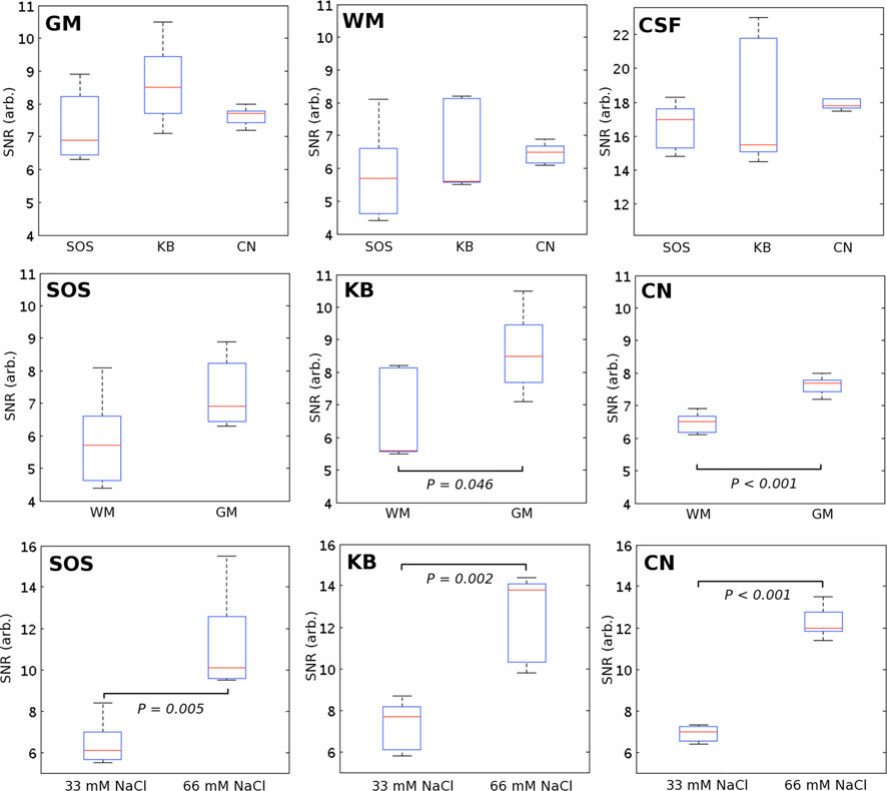
*Top row* shows SNR for GM, WM and CSF—grouped for the sequences to illustrate distribution. *Bottom rows* show pairwise results for WM, GM and 33/66 mM NaCl phantoms for SOS (*left*), KB (*middle*) and CN (*right*) to illustrate contrast between ROIs. *p*-values given when statistically significant (*p* < 0.05)

The upper part of Fig. 4 shows boxplots of the SNR results in GM, WM and CSF for the three sequences to illustrate the similarity of mean SNR. Differences in SNR between WM and GM were statistically significant for KB and CN (*p* = 0.046 and <0.001, respectively), but not for SOS (*p* = 0.1), for this sample size (middle row, Fig. 4). SNR differences between the 33 and 66 mM NaCl in 4 % agar were statistically significant for all three sequences (bottom row, Fig. 4). In general, largest inter-sequence variation between scans were found for the KB and SOS sequences, of up to 6 AU differences in signal intensity between scans for the 66 mM NaCl in agar phantom for SOS and of up to 8.5 AU in CSF for KB (Table 2). CN in comparison has a maximum difference of 2.1 AU for the 66 mM NaCl in agar phantom and of up to 0.7 AU for CSF between the different scans. This is reflected in the standard deviations of the group means, which are significantly smaller for CN than for SOS and KB (*p* = 0.016, corrected).

### Resolution measures

#### *x*–*y* Plane

**Fig. 5:**
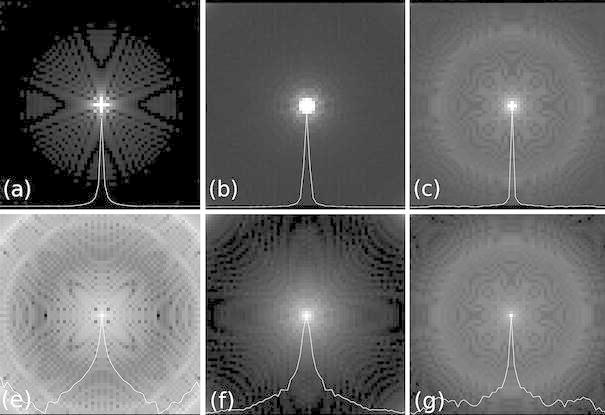
Image-space PSFs normalized between zero and one and shown at 10 % of their maximum intensities (**a–c**) and in logarithmic scale (**e**–**f**) for SOS (**a**, **e**), KB (**b**, **f**) and CN (**c**, **g**)

Figure 5 shows the image-space PSFs through the *x*–*y* plane, normalized to the same intensities, shown at 10 % of their maximum (top) and displayed on a logarithmic scale (bottom). Line profiles through the image center are shown overlaid on the images. At 10 % of the maximum intensity, SOS and KB have low background noise, whereas CN has a higher background noise level, its central linewidth is however the narrowest. Logarithmic scaling of the image as shown in the bottom row of Fig. 5 highlights the full extent of PSFs. Strong noise becomes apparent for SOS, whereas the noise in CN appears relatively unchanged. KB has a lower background noise profile at the cost of a wider central lobe. CN suffers diffuse background noise, but has the narrowest linewidth.

Compared to the theoretical PSF arising from Cartesian sampling, linewidths at FWHM are broadened by 0.32, 0.76 and 0.12 pixel for SOS, KB and CN respectively. At 10 % of the maximum as shown in the top of Fig. 5, the PSFs are broadened by 2.02, 2.12 and 0.2 pixel for SOS, KB and CN.

**Fig. 6:**
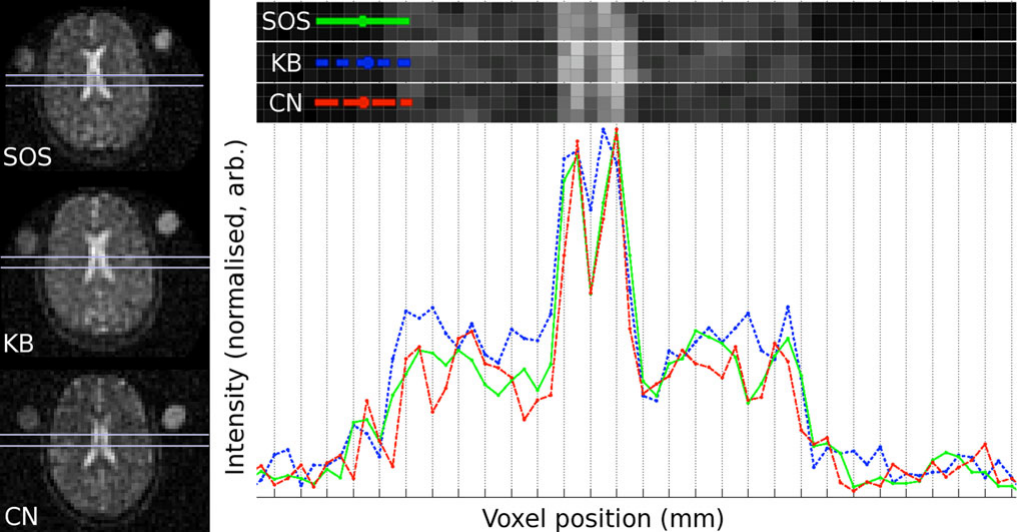
*Left column* shows a mid-transverse slice for one volunteer, showing the central ventricular area for SOS, KB and CN (*top* to *bottom*). Accentuated areas are of *three rows of pixels*, shown magnified in *top part* of the figure. Corresponding line profiles through the *central row of pixels* are shown underneath. *Line profiles* show wider and flatter peaks for the lateral ventricles in the KB-image, compared to SOS and CN. *Note* orientation and geometry is not entirely the same between the sequences, although great care was taken to match the anatomical structure on all three scans. Intensities have been normalized

Figure 6 shows the lateral ventricles for one volunteer, for all three sequences, as well as a close-up of three lines of pixels and a line profile through the anatomical structure. SOS and CN show the step from tissue to CSF similarly, albeit SOS peaks are slightly flatter and wider. Both SOS and CN have better defined peaks with linewidths that are narrower relative to KB, in agreement with the prediction from the theoretical PSFs.

#### *x*–*z* Plane

Stack-of-stars is encoded in the *z*-direction in a Cartesian way and has therefore no trajectory-based PSF broadening in this direction. The trajectories of KB and CN are roughly symmetric about the *z*-axis and their PSFs in the *x*–*z* plane are of the same appearance as those of the *x*–*y* plane shown in Fig. 5. They have small deviations in total linewidths however, compared to the PSFs of the *x*–*y* plane. PSF linewidths are broadened compared to the Cartesian PSF at FWHM by 0.76 and 0.08 pixel for KB and CN. At 10 % of the maximum, the PSFs are broadened by 2.2 and 0.12 pixel for KB and CN respectively.

#### Theoretical voxel volume

At FWHM, the PSF resulting from the Cartesian dataset is 1 pixel wide in the *x*, *y* and *z*-direction, assuming this to be the linewidth corresponding to a “real” 4 × 4 × 4 mm^3^ = 64 mm^3^ volume, the line broadening of SOS in the x and y direction results in a voxel volume of approximately 75 mm^3^. Likewise, considering the PSF broadening in the *x*, *y* and *z*-direction for KB and CN, their respective voxel volumes are 109 and 69 mm^3^.

#### Phantom measurements

**Fig. 7:**
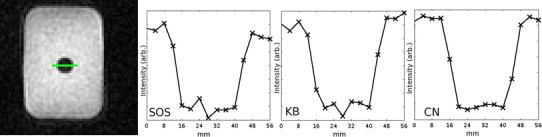
*Left image* shows the water-filled phantom (diameter 2.9 cm) suspended in saline solution (NaCl ≈ 155 mM). *Highlighted line* in the center of phantom representative of length of line profiles on the right. Distance between the top of the profile and the middle of the step-like profile was used as an approximation of the FWHM of the true image PSF. Measurements for resolution estimation were taken from both sides of the tube (as shown *left* and *right* in the line profiles) and at a perpendicular angle to these over three slices (not shown)

Figure 7 shows the phantom used for the resolution measurements and line profiles across it for the three sequences. Estimated actual resolution from the phantom was 8 ± 1, 9 ± 2.5 and 6.3 ± 2 mm for SOS, KB and CN respectively, taking four measurements across the phantom over three slices.

## Discussion

We have successfully adapted the CN sequence on a clinical MRI 3T system for ^23^Na-MRI in the brain and compared it to two established radial sampling schemes for SNR and resolution. For the sequence parameters used, all three sequences perform similarly in terms of SNR, but were found to differ in varying amounts from their prescribed resolutions, contrast and reproducibility.

The theoretical SNR differences are not different enough to generate statistically significant differences in measured SNR, although SOS follows the theoretical prediction with the lowest SNR values. Theoretically, KB has the least sampling uniformity and should therefore have a lower SNR efficiency, which has been offset with an optimized weighting function, as shown by the SNR efficiency calculated here. The higher measured SNRs for KB can also be explained in part by its broader PSF behaviour, which results in a true theoretical voxel volume that is around 1.5 times larger than for SOS and CN at FWHM and 1.1–1.35 times larger as measured from the resolution phantom. The larger voxel will increase SNR, as the larger volume will contain more MR-visible nuclei [11]. For example, the GM SNRs of 7.3, 8.6 and 7.6 AU for SOS, KB and CN respectively, would become respectively 6.2, 5.1 and 7.1 AU, and change if corrected for their theoretical voxel volumes of 75, 109 and 69 mm^3^ for the sequences respectively as before. As the definition of resolution through sequence parameters is very different and sequence-specific for non-Cartesian sequences, this also illustrates the problem of nominal- and “true”-resolution. Estimating the resolution directly from acquired images of the phantom follow the trend of the simulation, although with larger voxel sizes that are up to more than twice their prescribed value. Visual inspection of the brain images confirm the findings; the brain tissue in KB looks bright and homogenous, but flatter with less distinguished tissue edge details than in SOS and CN, as also seen in the line profiles through the lateral ventricles.

Variations in SNR between subjects are between ~5–45 % for KB and SOS, possibly due to different shim-quality and volunteer positioning, as well as minor physiological differences of the total sodium concentration between healthy volunteers. However, the variations are smaller for the CN sequence (~0–15 %), leading to an order of magnitude smaller total standard deviation, also suggesting that the larger variations in SOS and KB could be due to partial volume effects, which are less pronounced in CN. As CN was reconstructed using a NUFFT-method [30], different to the gridding used for SOS and KB, images from one volunteer for CN were reconstructed using a gridding method based on O’Sullivan [28] and results did not differ greatly between the images reconstructed through gridding or NUFFT, and noise was a normal distribution in both cases (not shown).

Comparison to previous studies is difficult, due to the variety of different field-strengths, scanner and coil hardware, sequences and sequence parameters used by different groups, as well as filtering and apodization used in post-processing that artificially enhance SNR, in addition to different methods in calculating SNR. Nagel et al. [24] reported SNR in brain tissue using a simple 3D-radial sequence to be ~7 AU, using a similar readout-length of 5 ms at a field strength of 3T.

Stack-of-stars and KB are simpler to program than CN, due to the more straightforward gradient waveform designs, but their shortcomings in true voxel size and reproducibility have to be evaluated when being used in clinical studies. Decreasing the voxel size of SOS and KB to match the true voxel size of CN would require increasing the scan time significantly, or require the use of more angular undersampling, which in turn would have negative consequences on their SNR, as well as increasing streaking artifacts, rendering them unsuitable for high resolution ^23^Na-MRI in clinically feasible scan times.

More sophisticated radial sequences, such as density adapted projection reconstruction [24] and sampling density-weighted apodization [33] (DA–PR and SDWA) have not been addressed in this study and could potentially overcome these shortcomings, as the gradient strength is adapted during sampling to yield more homogenously sampled *k*-space data, designed to increase SNR in ^23^Na-MRI. Cone-based trajectories, such as TPI and CN, due to their more efficient overall *k*-space coverage compared to basic radial sequences, require less number of readouts, therefore higher resolutions are achievable in less time with fewer undersampling artifacts, which make these sequences more suitable for high-resolution ^23^Na-MRI scanning at high-field strength.

## Conclusion

Two established UTE sequences for the detection of ^23^Na were compared against the CN technique. The CN sequence was found to offer better reproducibility and better partial volume behavior due to its narrow PSF, as compared to the radial sequences available to us, leading to an overall much improved spatial resolution, with minimal trade-down in SNR and total scan time, considering the differences in true voxel volume. Statistical analysis of the results demonstrate a shortcoming of SOS to distinguish the regional sodium difference between GM and WM for this sample size, with KB being borderline significant (*p* = 0.046). The difference in CN shows as highly significant (*p* < 0.001) and would therefore be a better choice for studies trying to identify small regional sodium concentration changes, as changes in total tissue sodium concentration are linked to SNR differences.

Having investigated three sequences from both a theoretical and practical perspective, results shown here will aid in the choice of sequence when attempting quantitative ^23^Na MRI at 3T in the human brain.
